# Brain gyrification in bipolar disorder: a systematic review of neuroimaging studies

**DOI:** 10.1007/s11682-022-00713-x

**Published:** 2022-08-31

**Authors:** Alessandro Miola, Giulia Cattarinussi, Maria Lavinia Loré, Niccolò Ghiotto, Enrico Collantoni, Fabio Sambataro

**Affiliations:** 1 Department of Neuroscience (DNS), University of Padova, via Belzoni 160, I-35121 Padua, Italy; 2 Padua Neuroscience Center, University of Padova, Padua, Italy

**Keywords:** Bipolar disorder, Gyrification, Neurodevelopmental disorders, Mania, Systematic review

## Abstract

Bipolar disorder (BD) is a severe mental illness with a strong genetic component. Genetic variations have been involved in the risk of this disorder, including those mediating brain function and neurodevelopment. Early neurodevelopment and neuroprogression processes could be reflected in brain gyrification patterns and help optimize the prediction and diagnosis of such disorders that is often delayed. Previous neuroimaging studies using this measure in patients with bipolar disorder revealed controversial results. This systematic review aimed to summarize available neuroimaging investigations on gyrification in BD compared to healthy controls (HC) and/or other psychiatric groups. Fourteen studies including 733 patients with BD, 585 patients with schizophrenia (SCZ), 90 with schizoaffective disorder (SZA), and 1380 healthy subjects were identified. Overall, a heterogeneous pattern of gyrification emerged between patients with BD and HC. Interestingly, increased gyrification or no differences were also observed in patients with BD compared to those with the schizophrenia-spectrum disorders. Furthermore, relatives of patients with BD showed lower or no differences in gyrification compared to healthy subjects without a family history of affective illness. Differences in the design and in methodological approaches could have contributed to the heterogeneity of the findings. The current review supports an altered brain gyrification pattern that underlies the pathophysiology of BD spanning large anatomical and functional neural networks, associated with altered cognitive functioning, difficulties in processing and affective regulation, and clinical symptoms. Longitudinal studies are needed to test different bipolar phenotypes and pharmacological effects on gyrification.

**Supplementary Information:**

The online version contains supplementary material available at 10.1007/s11682-022-00713-x.

## Introduction

Bipolar disorder (BD) is a clinically severe, episodic, lifelong mood disorder (Fagiolini et al., 2013). This disorder is associated with cognitive, behavioral, psychosocial, and functional impairment (Carvalho et al., 2020), reduced life expectancy, and higher risk of suicidal attempts (Anderson et al., 2012). According to the DSM-5, patients with BD experience mood states and energy swings between depression and emotional highs, which vary from manic to hypomanic episodes in BD type I (BD-I) or type II (BD-II), respectively (American Psychiatric Association, 2013).

Although the etiology of BD is largely unknown, some factors have been linked to the onset of this disorder. The heritability of BD ranges between 70 and 90% (Gordovez & McMahon, 2020) and it is hypothesized to follow mainly a common disease/common variant model with several genes, each with a small effect explaining the risk (Sullivan et al., 2012). Some of the risk genes, including *CACNA1C, CHRNA7, TCF7L2, NCAN, FGF-2,* and *MAPK1*, belong to pathways involved in neurodevelopment, neurodegeneration, neuroplasticity, and normal brain function in BD (Bem et al., 2019; Calabrò et al., 2016; Liu et al., 2014; Wang et al., 2018). Among them, previous evidence have highlighted the crucial role of Wnt signaling pathways underlying the pathogenesis of mood disorders, specifically for BD (Miola et al., 2022a; Sani et al., 2012), with one of its key enzymes, glycogen synthase kinase-3 beta, which regulates synaptic plasticity, cell survival, and circadian rhythms, involved in the pathophysiology and treatment of BD (Dandekar et al., 2018; Muneer, 2017). Furthermore, the Wnt pathway has also been reported as a regulator of crucial processes in the development of the mammalian nervous system and cortical gyrification (Chizhikov et al., 2019).

Several studies have shown that BD could be associated with neurodevelopmental alterations (Fornito et al., 2007; Sanches et al., 2008). Such variations, combined with unfavorable postnatal environmental factors during childhood and adolescence (Bortolato et al., 2017; O’Shea & McInnis, 2016) may lead to an early onset of BD with the first episode usually occurring around the age of 20 years (Carvalho et al., 2020). Indeed, adolescents who will develop BD may already show prodromal cognitive symptoms (Olvet et al., 2013), such as soft neurological symptoms (Mrad et al., 2016), which may be due to a deviation from the regular development trajectory. These changes are found more frequently in patients with early-onset BD (< 18 years) or BD with psychotic symptoms (Arango et al., 2014; Sigurdsson et al., 1999) that have had neurodevelopmental insults during fetal and early postnatal life (Canetta et al., 2014; Hozer & Houenou, 2016).

Gyrification is the process of brain remodeling of surface morphology to create sulci and gyri, thus expanding the cortical surface area (White et al., 2010). Cortical folding starts between 10 and 15 weeks of intrauterine life, reaches its peak during the third trimester of pregnancy (Sasabayashi et al., 2021), and continues until early infancy when it starts decreasing (White et al., 2010). Abnormalities of gyrification and of its trajectory are often associated with major psychiatric disorders, thus suggesting a role of aberrant neurodevelopment (Sasabayashi et al., 2021). Indeed, patients with psychiatric disorders may exhibit atypical primary gyri formation under neurodevelopmental genetic control in their fetal life and infancy, and then display higher-order gyral changes that are mainly due to mechanical stress derived from active brain changes rather than genetic load (Sasabayashi et al., 2021). Three hypotheses have been proposed to explain the gyrification process: The axons drawing highly interconnected regions of gray matter may cause tangential force components contributing to developing cortical folds (Essen, 1997); Reaction–diffusion mechanisms involving morphogens may cause a differential growth of sulci and gyri (Lefèvre & Mangin, 2010); Surface shell growing more than subcortical layers may result in a mechanical buckling that shapes the cortex (Toro & Burnod, 2005).

Thus, the gyrification index (GI), defined as the ratio between the perimeters of the pial and outer hull from structural *T*_*1*_*-*weighted magnetic resonance imaging (MRI) images (Zilles et al., 1988) could be particularly relevant for the analysis of the neurobiological changes underlying BD. GI can reflect both early alterations in neurodevelopmental trajectories and neuroprogression processes due to disorder-related mechanisms. Contrary to volume-based measures, which can be greatly influenced by the effects of age, illness process, and by the exposure to psychotropic treatment, cortical GI appears to be a more stable marker of neurodevelopment over time (Sasabayashi et al., 2021). The GI is generally computed on a slice-by-slice basis from a coronal projection in a two-dimensional space similarly to the approach used to calculate this index for post-mortem brains (Zilles et al., 1988). Since this approach could produce biased estimates in the case of slice orientation differences, three-dimensional surface-based or curvature-based measurements (3D-GI) have been proposed (Magnotta et al., 1999; Toro et al., 2008). GI can be computed as a global measure, calculated as the mean GI across the whole brain, as well as a regional measure, the local GI (LGI) (Schaer et al., 2008).

Unfortunately, previous studies employing this measure in BD revealed controversial results and the literature related to gyrification measures in this disorder has not been systematically evaluated so far. Therefore, this systematic review aimed to summarize available published MRI research reporting GI in patients with BD compared to healthy controls (HC) and/or other psychiatric groups.

## Methods

### Protocol and search strategy

This systematic review followed a pre-defined protocol available online (https://osf.io/54xcf/), and adhered to the procedures of the Preferred Reporting Items for Systematic Reviews and Meta-Analyses (PRISMA) statement (Moher et al., 2009) to ensure a high standard of reporting. A systematic search was conducted across two databases (PubMed and Scopus) without any language restriction to identify peer-reviewed articles published up to 5^th^ October, 2021. The search key was "(Gyrification or Gyrification index or GI) AND (bipolar disorder or Bipolar*)".

### Eligibility

Experimental, case–control, cross-sectional, and prospective or cohort study designs were considered eligible. Articles meeting the following criteria were included in this review:

- carried out in humans; b) reporting original findings; c) patients formally diagnosed according to the Diagnostic and Statistical Manual of Mental Disorders (DSM) or the International Classification of Diseases (ICD); d) using high-resolution MRI T1 weighted images; e) including at least a sample of patients with BD; f) evaluated gyrification as part of the analysis and reported the GI values of patients with BD.

### Data extraction

The authors independently screened the existing literature and extracted the data (A.M., M.L.L., N.G.), with a senior author solving disagreements and making the final decision (F.S.). Each article was required to pass a first- and second-level inspection to be included in this systematic review. This involved a title and abstract search and a full-text search, respectively. The following variables were extracted from a predefined excel spreadsheet: author, year, study design, country, sample size, age group, sex, diagnosis, patient care (inpatient vs. outpatient), duration of illness, diagnostic criteria, and diagnostic tools.

### Study quality and results

The quality of the included studies was assessed with the Imaging Methodology Quality Assessment Checklist (adapted from Strakowski et al, 2000) (Strakowski et al., 2000) (see Supplementary Material).

The R-package ggseg was used for the visualization of results in the Desikan–Killiany atlas (Mowinckel & Vidal-Piñeiro, 2020).

## Results

### Search results

**Fig. 1:**
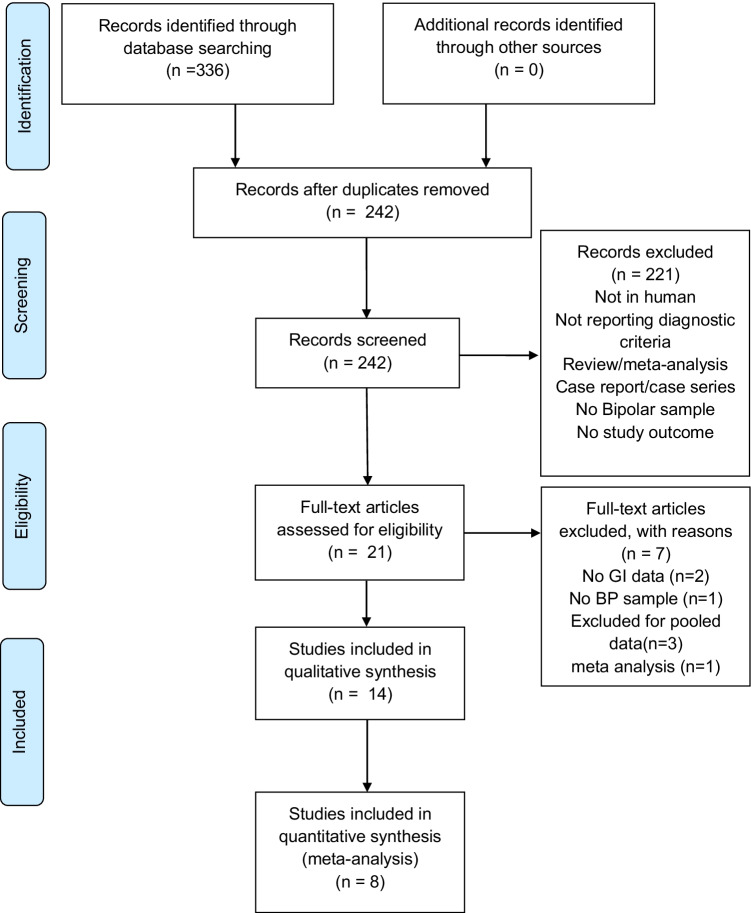
The Preferred Reporting Items for Systematic Reviews and Meta-Analyses (PRISMA) flow-chart

We identified 336 studies through our electronic search; no additional references were found from other sources. After removing duplicates, the title and abstract of 242 papers were screened, and 221 were excluded. The reasons for the exclusion of the latter reports are summarized in the appropriate box provided in Fig. 1. We evaluated the full text of 21 papers and excluded 7 additional references for different reasons according to Fig. 1 and specified in the list of excluded studies in Table S2. A total of 14 studies were included in our review.

### Characteristics of the included studies

Fourteen studies involved 733 patients with BD (35.5 ± 11.9 years; 51.6% female), 29 relatives of patients with mixed BD-major depressive disorder (MDD, 21.3 ± 4.1 years; 65.5% female), and 1380 healthy controls (HC, 33.4 ± 12.3 years; 47.1% female) were included in this systematic review (see Table 1 for details). The BD-MDD group was composed of 29 offspring of BD with a lifetime diagnosis of affective disorders with one parent diagnosed with a primary mood disorder. In this group, relatives of patients with MDD were also recruited because it is estimated that about 70% of depressed first-degree relatives of BD are affected with BD and because the episode of the depressive index usually precedes the onset of BD (Drobinin et al., 2019). Data from papers with other psychiatric samples were included if a direct comparison of GI with the BD sample was made. This selection resulted in nine papers with 585 patients diagnosed with schizophrenia (SCZ) (35.9 ± 10.2 years; 31.9% female), and one paper with 90 patients diagnosed with schizoaffective disorder (SZA) (35.7 ± 12.2 years; 56% female) (Nanda et al., 2014). The majority of studies did not report the mood state of the patients at the time of the scan. Ten studies specifically reported that patients with BD received medications at the time of scan (Choi et al., 2020; Drobinin et al., 2019; Janssen et al., 2014; Madeira et al., 2020; Madre et al., 2020; Mirakhur et al., 2009; Nanda et al., 2014; Nenadic et al., 2015; Palaniyappan & Liddle, 2014; Palaniyappan et al., 2019). Seven studies enrolled patients with BD-I, while the others did not specify whether BD-I or II was included. Choi et al. (2020) explored GI in patients with BD-I and BD-II and conducted a subgroup analysis comparing clinical samples (Choi et al., 2020). Three studies included patients with BD with psychotic symptoms (Janssen et al., 2014; Palaniyappan & Liddle, 2014; Palaniyappan et al., 2019). Additionally, two studies investigated a group of first-degree relatives of patients with BD (Drobinin et al., 2019; Nanda et al., 2014). One study had a longitudinal design (Mirakhur et al., 2009) and another paper evaluated the trajectories of changes in gyrification over the course of the disorder (Cao et al., 2017a).

**Table 1:** Demographic and characteristics of included studies

First author, study year	Diagnostic criteria	Diagnosis	Population setting	Mood state	Illness duration (years)	BD N (% F) [age mean ± SD, years]	Comparison group Diagnosis, N (% F) [age mean ± SD, years]	HC Cohort, N (% F) [age mean ± SD, years]	Quality score
Janssen et al., 2014	DSM IV-TR	EOP BD-I with psychotic symptoms	Outpatients, inpatients	NR	NR	20 (35) [16.4 ± 1.6]	SCZ, 20 (10) [15.8 ± 1.8]	52 (39.5) [15.4 ± 1.5]	10
Cao et al., 2017a	DSM-IV, SCID I	BD-I	NR	7% mania, 46% depression 22% euthymia	NR	69 (NR) [NR]	NR	80 (NR) [NR]	7.5
Mirakhur et al., 2009	DSM-IV (SCID); OPCRIT	BD-I	NR	NR	14.3 ± 8.5	18 (56) [38.4 ± 8.4]	NR	18 (50) [36.7 ± 13.2]	10
Liao et al., 2008	(DSM-IV)	BD-I	Outpatients	NR	9 ± 7	24 (58) [36 ± 11]	NR	24 (NR) [NR]	8.5
Madre et al., 2020	DSM-IV	BD	NR	NR	14 ± 10	128 (42.2) [41 ± 10]	SCZ, 128 (42.2) [41 ± 10]	127 (42.5) [39 ± 10]	10
Palaniyappan et al., 2019	DSM-IV	BD with psychotic symptoms	NR	NR	11.05 ± 8.13	22 (36.4) [34.6 ± 10.4]	SCZ, 34 (14.7) [32.9 ± 8.9]	NR	10
Choi et al., 2020	DSM-IV-TR	BD	Outpatients	64% euthymia 36% depression	49.8 ± 66.7	61 (45.9) [33.3 ± 11.4]	NR	183 (57.4) [33.5 ± 13.3]	10
Palaniyappan & Liddle, 2014	DSM-IV	BD with psychotic symptoms	Outpatients	NR	NR	20 (NR) [NR]	SCZ, 39 (NR) [NR]	34 (NR) [NR]	9
Drobinin et al. 2019	DSM-IV	BD	Outpatients	NR	4.6 ± 2.9	29 (65.5)* [21.3 ± 4.1]	UR, 32 (66) [19.3 ± 3.1]	42 (60) [22.3 ± 4.1]	9.5
Cao et al., 2017b	DSM-IV	BD-I	NR	NR	NR	151(55) [32.1 ± 16.1]	MDD, 95 (63.2) [36.5 ± 16.5] SCZ, 125 (21.6) [38.4 ± 13]	SA, 225 [27.7 ± 14.2] NKI, 139 [33.6 ± 20.1] COBRE, 146 [38.5 ± 11.5]	9.5
Nanda et al. 2014	DSM-IV	BD with psychotic symptoms	NR	NR	NR	141 (69) [36.6 ± 13]	SZA, 90 (56) [35.7 ± 12.2] SCZ, 157 (36) [34.3 ± 12.2] FDR, 300 (71) [39.8 ± 16.1]	243 (53) [37.5 ± 12.3]	10
Nenadic et al., 2015	DSM-IV, DSM-III-R	BD-I	Outpatients, inpatients	Euthymic	NR	17 (47) [37.7 ± 11.1]	SCZ, 34 (38.2) [33.0 ± 8,9]	34 (47) [34.3 ± 10.6]	9
McIntosh et al., 2009	OPCRIT, DSM-IV (SCID)	BD-I	Outpatients	Euthymic	NR	42 (52.4) [39.6 ± 10.1]	SCZ, 28 (46.4) [38 ± 9.9]	37 (51.3) [38.4 ± 10.7]	9
Madeira et al., 2020	ICD-10	BD	Inpatients	NR	5.2 ± 4.3	20 (35) [31.6 ± 10]	SCZ, 20 (35) [31.5 ± 10.3]	20 (35) [31.5 ± 10.3]	10

*BD* bipolar disorder; *BD-I* bipolar disorder type I; *, affected relatives; *UR* unaffected relatives; *FDR* first-degree relatives, *SCZ* schizophrenia; *SZA* schizoaffective disorder; *EOP* early-onset first-episode psychosis; *OPCRIT* Operational Criteria symptom checklist; *COBRE* Center of Biomedical Research Excellence; *HC* healthy controls; *MDD* major depressive disorder; *NKI* Nathan Kline Institute; *SA* San Antonio; *F* female. Quality score was assessed using the Imaging Methodology Quality Assessment Checklist

### Gyrification in BD compared to HC

#### Methodological characteristics

Among the studies that compared patients with BD and HC, four used a region of interest (ROI) approach and ten performed a whole-brain analysis. In general, the results were heterogeneous due to different study protocols and methodological approaches, with seven investigations that did not reveal differences in cortical gyrification between samples (Cao et al., 2017a; Drobinin et al., 2019; Janssen et al., 2014; Liao et al., 2008; Madre et al., 2020; Mirakhur et al., 2009; Nanda et al., 2014). Negative results were also shown by cross-sectional studies using LGI (Madre et al., 2020) and 3D-GI measures (Liao et al., 2008).

#### Severity

Janssen et al. (2014) compared patients with BD with psychotic symptoms and early onset first episode SCZ with HC and found a difference in frontal GI in patients compared to HC, but this result did not survive multiple comparisons correction (Janssen et al., 2014). Similarly, a study conducted in BD-I showed a decreased GI in patients with more than three manic episodes compared to HC, although this result did not survive multiple comparison correction (Cao et al., 2017a).

#### Design

A longitudinal investigation that followed participants over the course of four years showed a similar rate of decrease in ventral and dorsal prefrontal GI in BD and HC. No differences in GI were reported in patients (Mirakhur et al., 2009).

#### Familiarity

Similarly, Nanda et al. (2014) found altered gyrification in psychosis and yet failed to identify LGI differences in probands with psychotic BD compared to healthy subjects (Nanda et al., 2014).

**Fig. 2:**
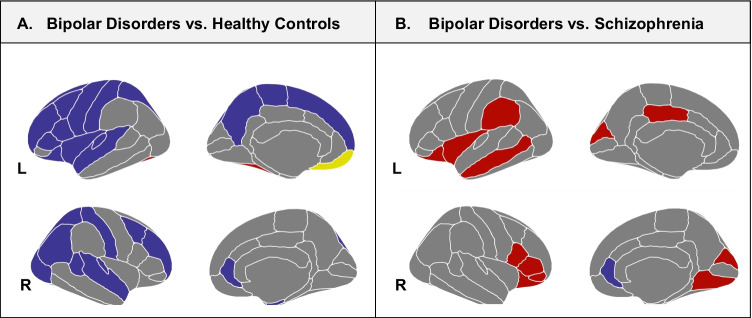
Gyrification alterations in studies in bipolar disorders. The brain regions showing gyrification differences in studies in patients with bipolar disorders compared to healthy controls (**A**) or schizophrenia (**B**) are displayed in the Desikan–Killiany atlas. Red indicates regions where studies report increased gyrification in bipolar disorders; blue indicates where gyrification is decreased; yellow indicates when the results are mixed. L, left hemisphere; R, right hemisphere. Medial and lateral surfaces are displayed for each hemisphere. The renderings were created using the R-package *ggseg*

Four investigations reported significant differences in GI between BD and HC (Table 2). In particular, Palaniyappan and Liddle (2014) revealed a reduced GI in BD with psychotic symptoms compared to HC involving the right and left middle frontal gyrus (MFG), the superior parietal gyrus (SPG), the postcentral gyrus, the lateral occipital gyrus and the precuneus, and an increased GI encompassing the left fusiform and left medial orbitofrontal gyri (OFG) in BD compared to HC (Palaniyappan & Liddle, 2014). Choi et al. (2020) showed hypogyria in the left pars opercularis, precentral gyrus, postcentral gyrus, superior temporal gyrus (STG), insula, right entorhinal cortex, and transverse temporal cortices in a sample of patients with BD during the euthymic or depressive phases compared to HC. No differences in cortical GI between BD subtypes were reported. In addition, GI values did not correlate with the duration of the illness or the severity of depression (Choi et al., 2020). Consistently, McIntosh showed a reduction in prefrontal GI in BD (McIntosh et al., 2009). In contrast, Nenadic et al. (2015) observed an increase in GI in patients with BD in the right anterior infra-genual cingulate cortex (CC) and the left dorsolateral prefrontal cortex (DLPFC) (Nenadic et al., 2015). The results of the alterations in gyrification in BD compared to HC are shown in Fig. 2A.

**Table 2:** Summary of the structural imaging findings of the studies reported on GI measures

First Author, year	Study design	Country	Field Strength (Tesla)	Imaging Software	Gyrification measure	Space/Atlas	Analysis	Main findings
Janssen et al., 2014	cross-sectional	Spain	1.5	Freesurfer 5.1	Lobar GI	Native space	Whole brain	BD and SCZ vs HC: ↓CT and ↑ SW in the frontal lobe; ↓ GI in the frontal (did not survive correction for multiple comparisons). Negative correlation between SW and GI SCZ vs HC: ↑ SW in all frontal, temporal, parietal, and occipital lobes No correlations between morphometry and cumulative antipsychotic dose
Cao et al., 2017a	cross-sectional	USA	1.5	Freesurfer 5.3.0	Local GI	NA	Whole brain	BD (> 3 manic episodes) vs HC: ↓ GI (not surviving Bonferroni correction) Negative correlation between GI and progression BD
Mirakhur et al., 2009	longitudinal	Scotland	1.5	NA	Local GI	NA	ROI (ventral and dorsal Prefrontal)	BD-I vs. HC: ↓ ventral and dorsal GI after 4 years with a similar rate of change BD with ≥ 1 vs BD with < 1 BDNF met allele: ↓ rate of change in GI that correlated with ↓ GI in the left hemisphere No correlation between the rate of change in GI and medication
Liao et al., 2008	cross-sectional	Taiwan	1.5	SPM5	(3-D) GI	MNI/AAL	Whole brain	BD vs HC: no significant differences for 3-D GI; significant changes in the asymmetry of limbic lobe CC in BD. Trends of significance for difference of 3-D GI and asymmetry of CC in the frontal lobe Negative correlation between asymmetry coefficients in the frontal lobe and HAMD-17 and HARS scores and a trend for significance for MADRS and YMRS scores Negative correlation between the duration of illness and GI in the right limbic lobe
Madre et al., 2020	cross-sectional	Spain	1.5	FreeSurfer	Local GI	MNI	Whole-brain	BD and SCZ vs HC: widespread ↓ CV and CT in similar areas BD vs SCZ: ↑ in CV, CT, SA and GI. Differences in GI included a cluster in the left supramarginal gyrus and bilateral cuneus BD vs HC: no areas of SA or GI abnormalities
Palaniyappan et al., 2019	cross-sectional	UK	3	FreeSurfer 5.1.0	Local GI	NA	Whole brain	BD and SCZ: trend for a significant positive correlation between cortical GI and speech connectedness
Choi et al., 2020	cross-sectional	UK	3	FreeSurfer 5.3	Local GI	MNI/Desikan–Killiany atlas	Whole brain	BD vs. HC: ↓ GI in the left inferior frontal gyrus (pars opercularis), precentral gyrus, postcentral gyrus, superior temporal cortex, insula, right entorhinal cortex, and transverse temporal cortices No correlations between GI and clinical factors, including illness duration, depressive symptom severity, and lithium treatment No significant differences in cortical GI between BD subtypes
Palaniyappan & Liddle, 2014	cross-sectional	UK	3	FreeSurfer 5.1.0	Local GI	MNI	Whole-brain	BD vs SCZ: ↑ GI in right lingual, left posterior cingulate, and bilateral orbital fronto-insular regions BD vs HC: ↓ GI in right caudal and rostral middle frontal, right superior parietal, postcentral, and lateral occipital gyri, and left middle frontal and precuneus. ↑ GI in left fusiform and left medial orbitofrontal gyri
Drobinin et al., 2019	cross-sectional	UK	1.5	FreeSurfer 5.3	3D Local GI	MNI/Desikan–Killiany atlas	ROI (IFG)	UR and AR vs HC: rIFG (par triangularis) volume UR > AR > HC Mean SA of the pars triangularis UR = AR > HC No correlations between pars triangularis volume and GI No differences in GI or CT of the pars triangularis
Cao et al., 2017b	cross-sectional	USA	1.5	FreesSurfer 5.3	Local GI	NA	Whole-brain	BD and SCZ vs HC: faster ↓ in GI in multiple brain regions after 40 years. The GI decrease in BD-I was less extensive compared to SCZ
Nanda et al., 2014	cross-sectional	USA	3	FreeSurfer	Local GI	NA	Whole-brain	SCZ and SZA vs HC: ↓ GI in the right posterior cingulate, right caudal anterior cingulate and left caudal anterior cingulate FDR with Axis II cluster A disorders vs HC: nearly significant ↓ GI in these same regions SCZ vs SZA vs BD vs HC: SCZ < HC, anterior cingulate GI; SZA < HC, left pars opercularis, right inferior parietal, right banks of the superior temporal sulcus and right superior temporal; BD vs HC, BD vs SZ, no differences in GI
Nenadic et al., 2015	cross-sectional	Germany	3	Freesurfer	Local GI	NA	ROI (DLPFC, ACC and medial PFC)	BD-I vs HC: ↑ GI in the right anterior infra-genual cingulate and left DLPFC SCZ vs HC: ↑ GI in the right anterior medial prefrontal cortex and orbitofrontal cortex BD-I vs SCZ: ↑ GI in the right anterior infra-genual cingulate
McIntosh et al., 2009	cross-sectional	UK	1.5	SPM	Lobar GI	MNI/Desikan-Killiany Atlas	ROI (ventral and dorsal prefrontal)	BD vs SCZ vs HC: ↓ prefrontal GI compared with HC, no differences with SCZ Positive correlation between IQ and GI
Madeira et al., 2020	cross-sectional	Portugal	3	CAT12	Local GI	MNI	Whole-brain	BD vs SCZ vs HC: BD > HC > SCZ, GI in the left supramarginal gyrus SCZ vs BD and HC: BD = HC > SCZ in the right inferior frontal gyrus BD: positive correlation between GI in the left supramarginal gyrus and antipsychotic dosage BD and SCZ: negative correlation between GI in the left supramarginal gyrus and scores on the Schizo-Bipolar Scale and positive correlation with functioning; negative correlation between GI in the right inferior frontal gyrus and antipsychotic dosage

*AAL* Anatomical Automatic Labeling; *SA* surface area; *GI* gyrification index; *CC* cortical complexity; *CV* cortical volume; *CT* cortical thickness; *SW* sulcal width; *BD* bipolar disorder; *BD-I* bipolar disorder type I; *SCZ* schizophrenia; *SZA* schizoaffective disorder; *AR* affected relatives; *UR* unaffected relatives; *FDR* first-degree relatives; *HC* healthy controls; *BA* Brodmann Area; *IQ* intelligent quotient; *BDNF* Brain-derived neurotrophic factor; *HAMD-17* 17-item Hamilton Rating Scale for Depression; *HARS* Hamilton Anxiety Rating Scale; *MADRS* Montgomery-Asberg Depression Rating Scale: *YMRS* Young Mania Rating Scale. *IFG* inferior frontal gyrus; *DLPFC* dorsolateral prefrontal cortex; *ACC* anterior cingulate cortex; *PFC* prefrontal cortex

### Gyrification in BD compared to other psychiatric disorders

#### SCZ

Madre et al. (2020) observed an increase in GI in a cluster encompassing the left supramarginal gyrus and bilateral cuneus in BD compared to SCZ (Madre et al., 2020). The same research group conducted two studies on cortical gyrification in BD and SCZ (Palaniyappan & Liddle, 2014; Palaniyappan et al., 2019). The first showed higher GI in right lingual, left posterior cingulate (PCC), and bilateral orbital fronto-insular regions in BD compared to SCZ (Palaniyappan & Liddle, 2014), while the second explored the association between GI and speech connectedness, a structural feature aimed at measuring the loosening of associations, calculated using non-semantic graph analysis in a sample of BD and SCZ and showed a trend for a significant positive correlation between cortical GI and speech connectedness in SCZ (Palaniyappan et al., 2019). Conversely, hypergyria in the right anterior infra-genual CC (Nenadic et al., 2015), as well as in the left supramarginal gyrus was reported in BD compared to SCZ (Madeira et al., 2020). The results are shown in Table 2 and Fig. 2B.

#### SCZ and MDD

Cao et al., 2017b) observed abnormal gyrification trajectories in a large cross-sectional sample with a 4–83 age range of patients with major psychiatric disorders (BD, SCZ, MDD) compared to healthy individuals. Specifically, GI was significantly lower in multiple brain regions and less extensive in patients with BD-I and SCZ than HC, and became manifest after the age of 40 years (Cao et al., 2017b).

#### SCZ and SZA

Nanda et al. (2014) and McIntosh et al. (2009) did not show significant GI differences in BD relative to SZA and SCZ, respectively (McIntosh et al., 2009; Nanda et al., 2014).

### Gyrification in relatives of BD

Two studies explored GI in relatives of BD probands (Drobinin et al., 2019; Nanda et al., 2014). *BD.* A volumetric and surface ROI analysis of the inferior frontal gyrus (IFG) was performed in affected and unaffected offspring of BD and HC. Although volumetric and surface area differences were found, gyrification did not differ between groups (Drobinin et al., 2019) (see Table 2).

#### SCZ-BD spectrum

In a mixed sample of first-degree relatives of patients with psychosis within the schizophrenia-bipolar spectrum, Nanda and colleagues (2014) found a trend of significance for hypogyria in the right PCC and bilateral caudal anterior cingulate (ACC) compared with control subjects (Nanda et al., 2014). Unfortunately, the small sample size of BD relatives (N = 5) limits the generalizability of this finding.

### Associations between psychotropic treatments and gyrification

A total of seven studies explored the effect of medications on GI (Choi et al., 2020; Janssen et al., 2014; Madeira et al., 2020; Mirakhur et al., 2009; Nanda et al., 2014; Palaniyappan & Liddle, 2014; Palaniyappan et al., 2019). No correlations were found between GI and antipsychotics (Janssen et al., 2014; Nanda et al., 2014; Palaniyappan & Liddle, 2014; Palaniyappan et al., 2019), lithium (Choi et al., 2020), or the dosage of mood stabilizers (Palaniyappan & Liddle, 2014). Consistently, no association was observed between exposure to four different classes of psychotropic medications (lithium, typical antipsychotics, mood stabilizers, and antidepressants) and the rate of change in GI in BD after 4 years (Mirakhur et al., 2009). Differently, a positive correlation between GI in the left supramarginal gyrus and antipsychotics emerged in BD (Madeira et al., 2020), as well as between GI values and lithium treatment in the right ACC and right PCC (Nanda et al., 2014).

## Discussion

In this paper, we systematically examined all the existing literature on gyrification abnormalities in BD. In general, a heterogeneous picture of decreased, increased, or similar gyrification between BD and HC emerged. Seemingly, an increase or no differences in GI were observed in BD relative to SCZ or SZA, while relatives of BD showed lower or similar GI compared to healthy subjects without a family history of BD.

Regarding the comparison between BD and HC, the results are mixed and heterogeneous. Some studies did not find abnormalities in cortical GI (Cao et al., 2017a; Drobinin et al., 2019; Janssen et al., 2014; Liao et al., 2008; Madre et al., 2020; Mirakhur et al., 2009; Nanda et al., 2014), while others reported significant regional differences (Choi et al., 2020; McIntosh et al., 2009; Nenadic et al., 2015; Palaniyappan & Liddle, 2014). Interestingly, from a topological point of view, the findings of altered gyrification are widespread. Indeed, reductions in GI were observed in the frontal (Choi et al., 2020; McIntosh et al., 2009; Palaniyappan & Liddle, 2014), parietal (Choi et al., 2020; Palaniyappan & Liddle, 2014), temporal (Choi et al., 2020) and occipital lobes (Palaniyappan & Liddle, 2014). Furthermore, an increased GI in BD was described in CC and DLPFC (Nenadic et al., 2015), temporal and OFG (Palaniyappan & Liddle, 2014), as well as in the left supramarginal gyrus and bilateral cuneus (Madre et al., 2020). These observations support the evidence that the pathogenesis of BD is characterized by an alteration of large anatomical and functional neural networks, which span across the default mode, the cognitive control, and the salience networks. Interestingly, changes in local gyrification have been suggested to represent an indirect sign of altered underlying connectivity in the corresponding areas, which in turn appears to be associated with changes in cognitive functioning and clinical symptoms (White & Hilgetag, 2011). In particular, our results showed a pattern of abnormalities in BD in areas encompassing the fronto-parietal cognitive control network, a brain circuit involved in several executive functions, including attention, planning, and working memory (Breukelaar et al., 2016). As deficits in these cognitive domains have been commonly reported in BD (Bora, 2018), we could hypothesize that abnormalities in gyrification could be involved in the development of cognitive alterations in BD. In addition, we also found differences in GI in areas included in the default mode and the salience networks, particularly in the precuneus, the ACC, the DLPFC, and the insula, possibly underlying deficits in the processing and regulation of affective stimuli (Rai et al., 2021).

Cortical gyrification patterns in BD were also compared with other psychiatric disorders, including SCZ and SZA that are thought to be associated with alterations in neurodevelopmental trajectories (Parellada et al., 2017), and gyrification may represent an important and relatively stable biomarker of neurodevelopment (Sasabayashi et al., 2021). Neurodevelopmental deviations, including neuromotor and cognitive impairment, although marked and severe in SCZ (Betts et al., 2016; Cannon et al., 2002; MacCabe et al., 2008; Parellada et al., 2017; Seidman et al., 2013) are also present in BD. Several regions, including the cingulate, insular, frontal, parietal, and occipital cortices, showed an increase in LGI in BD relative to SCZ (Madeira et al., 2020; Madre et al., 2020; Nenadic et al., 2015; Palaniyappan & Liddle, 2014) and only two studies failed to show any difference in this measure (Nanda et al., 2014; Nenadic et al., 2015),

Overall, these studies support the presence of greater alterations in GI in SCZ in large-scale brain networks, which could be due to the presence of more pronounced early developmental alterations or more dramatic neuroprogression phenomena in SCZ (Palaniyappan et al., 2013a, b; Sasabayashi et al., 2021) and could result in cognitive and affective symptoms in this population (Orliac et al., 2013). The current literature does not allow us to clearly distinguish whether gyrification abnormalities occur before the onset of BD or if they result from neuroprogression mechanisms. Furthermore, although gyrification is considered rather stable during brain development, it has been shown that GI changes quite rapidly in response to variations in nutritional status or hydration (Bernardoni et al., 2018). Since patients with BD are subject to even very rapid fluctuations in these parameters (Bernardoni et al., 2018), future research should consider these parameters.

Lastly, the role of GI as a potential biomarker of risk for BD remains unclear. Among the two studies that explored GI in relatives of BD, the first did not report differences between affected and unaffected offspring of BD and HC without a family history of BD in the IFG (Drobinin et al., 2019), a region previously proposed as a candidate endophenotype for BD (Cattarinussi et al., 2019), while the second showed a nearly significant reduction in GI in first-degree relatives of BD with cluster A personality disorder compared to HC (Nanda et al., 2014). These results should be interpreted in light of some factors: first, the sample of the study by Drobinin et al. (2019) was relatively small and included young adults, while Nanda et al. (2014) explored GI in a considerably larger and older sample of relatives (mean age = 39.8 ± 16.1 years) (Nanda et al., 2014). Furthermore, the investigation by Nanda et al. (2014) observed differences in GI only between relatives of the schizophrenic bipolar spectrum with cluster A personality disorder, with a small number of relatives of BD and HC, while they reported no differences between the general sample of relatives and HC, suggesting that the decrease in GI in relatives could be associated with the presence of a cluster A personality disorder (Nanda et al., 2014). Overall, these studies did not show alterations in GI in unaffected first-degree relatives, while the picture was mixed for affected relatives. Since volumetric alterations in affected and unaffected relatives of BD have been consistently reported (Cattarinussi et al., 2019, 2022), future studies with larger sample sizes should explore cortical measures in relatives to clarify their possible role in increasing the risk of the disorder. Indeed, as a more stable neurodevelopmental marker compared to gray matter volume-based measures (Sasabayashi et al., 2021), the brain gyrification pattern could provide a promising biomarker potentially useful for early identification of BD in those individuals at-risk mental state (Sasabayashi et al., 2017).

Unfortunately, only one study explored GI in BD-I and BD-II and found no differences (Choi et al., 2020). Since BD subtypes have distinct clinical and epidemiological features, the study of cortical measures, particularly gyrification, could help in exploring possible underlying differences in neurodevelopment. In this regard, larger studies on BD subtypes are needed.

Our qualitative synthesis presents highly heterogeneous findings as a result of different methodological approaches and different BD phenotypes. From a methodological perspective, some studies reported GI using an ROI-based approach (Drobinin et al., 2019; McIntosh et al., 2009; Mirakhur et al., 2009; Nenadic et al., 2015), while others conducted whole-brain analyses (Cao et al., 2017a, b; Choi et al., 2020; Janssen et al., 2014; Liao et al., 2008; Madeira et al., 2020; Madre et al., 2020; Nanda et al., 2014; Palaniyappan & Liddle, 2014; Palaniyappan et al., 2019). Furthermore, most MRI investigations used a cross-sectional design (Cao et al., 2017a, b; Choi et al., 2020; Drobinin et al., 2019; Janssen et al., 2014; Madeira et al., 2020; Madre et al., 2020; McIntosh et al., 2009; Nanda et al., 2014; Nenadic et al., 2015; Palaniyappan & Liddle, 2014; Palaniyappan et al., 2019), and only one longitudinal study (Mirakhur et al., 2009) has been conducted. Furthermore, some works were performed using 1.5 T MRI (Cao et al., 2017a, b; Drobinin et al., 2019; Janssen et al., 2014; Liao et al., 2008; Madre et al., 2020; McIntosh et al., 2009; Mirakhur et al., 2009), while other studies were conducted with 3 T MRI scan (Choi et al., 2020; Madeira et al., 2020; Nanda et al., 2014; Nenadic et al., 2015; Palaniyappan & Liddle, 2014; Palaniyappan et al., 2019), as already reported for other surface-based measures (Han et al., 2006). Lastly, some of the studies included young patients with BD (Cao et al., 2017b; Janssen et al., 2014), while others focused on adult BD. In addition, the clinical characteristics of patients with BD were highly heterogeneous. Some studies reported GI findings among patients with BD without specifying whether BD-I or BD-II patients were included (Choi et al., 2020; Drobinin et al., 2019; Madeira et al., 2020; Madre et al., 2020; McIntosh et al., 2009; Nanda et al., 2014; Palaniyappan & Liddle, 2014), while others focused specifically on BD-I (Cao et al., 2017a, b; Liao et al., 2008; McIntosh et al., 2009; Mirakhur et al., 2009), BD with psychotic symptoms (Nenadic et al., 2015; Palaniyappan et al., 2019), and first episode BD-I with psychotic symptoms (Janssen et al., 2014). Additionally, only a few studies specified the mood state of the patients with BD (Cao et al., 2017a; Choi et al., 2020; Janssen et al., 2014; Liao et al., 2008; Madeira et al., 2020; McIntosh et al., 2009; Nenadic et al., 2015). After this, four studies included outpatients (Choi et al., 2020; Drobinin et al., 2019; Liao et al., 2008; McIntosh et al., 2009), and two reports explored inpatients with BD (Madeira et al., 2020; Palaniyappan & Liddle, 2014), two investigations were carried out in a mixed population setting of BD (Janssen et al., 2014; Nenadic et al., 2015), and for six the setting was not specified (Cao et al., 2017a, b; Madre et al., 2020; Mirakhur et al., 2009; Nanda et al., 2014; Palaniyappan et al., 2019). Only half of the included studies provided details about the duration of illness of patients with BD (Choi et al., 2020; Drobinin et al., 2019; Liao et al., 2008; Madeira et al., 2020; Madre et al., 2020; Mirakhur et al., 2009; Palaniyappan et al., 2019), with previous MRI studies revealing a negative correlation between GI and stages of BD (Cao et al., 2017a) and a negative correlation between the duration of illness and GI in the right limbic lobe (Liao et al., 2008).

BD is characterized by a high rate of medical comorbidities, including obesity, type 2 diabetes, metabolic syndrome (Kemp et al., 2010; Miola et al., 2022a, 2022b; Sinha et al., 2018; Sylvia et al., 2015), as well as comorbid psychiatric disorders such as anxiety, substance or alcohol use, and eating disorders that can also occur before the onset of BD and act as confounders or can alter neurodevelopment in patients with BD (McElroy et al., 2001; Nabavi et al., 2015). Only a few studies reported comorbid conditions (Cao et al., 2017a; Palaniyappan & Liddle, 2014), thus hampering the possibility of exploring the effects of specific comorbidities on GI.

Notwithstanding the heterogeneity of findings that limits the potential clinical impact of the GI evaluation in BD, the implementation of such a measure could represent a valuable tool not only for the differential diagnosis of bipolar-spectrum disorders but also for the prediction of treatment response in BD. Indeed, GI has been shown to be a useful predictor of antipsychotic treatment response for both affective and nonaffective psychoses, with nonresponders showing prominent hypogyria in the bilateral insular, left frontal and right temporal regions compared to responders (Palaniyappan et al., 2013b).

This systematic review presents some limitations that must be acknowledged. First, the cross-sectional design of most included studies limits the generalizability of our results and our ability to make inferences about the causality of neurodevelopmental alterations and BD. Second, differences in the estimation of gyrification, whole brain vs. regional approaches, and field strength of the MRI scanners could have contributed to the heterogeneity of the findings. Third, the demographics of the sample, including age and sex, in addition to medical and psychiatric comorbidities, could partly explain these mixed findings. Fourth, in the included investigations, no information was reported on early life adverse events or childhood adversities, which may be associated with structural alterations in the brain of patients with BD (Janiri et al., 2019; Zovetti et al., 2022). Fifth, only a few studies evaluated the effect of medications on GI. Again, given the paucity of studies that have longitudinally tested the effects of medication use on the morphology of gyrification, it is difficult to assess the role of medications as confounders of gyrification over time. Moreover, it remains a challenging to unravel the effects of the disease itself, neuroprogression, and chronicity. Future studies are needed to better clarify these aspects.

## Conclusions

Overall, although clinical implications are still limited due to the heterogeneity of current findings, this review provides the current state of the art on the role of brain gyrification in the pathophysiology of BD. Despite some inconsistencies in the results, our review showed a pattern of altered gyrification in BD in large neural networks, spanning from the default mode, the cognitive control, and the salience networks, which could result in impaired cognitive functioning, altered emotional regulation, and clinical symptoms. Future longitudinal studies in subjects at high-risk of BD that incorporate gyrification measures to refine bipolar phenotypes and test the effects of genes associated with BD are warranted.

## Supplementary Information

Below is the link to the electronic supplementary material.

Supplementary file1 (DOCX 1206 KB)

## Data Availability

Data supporting the findings of this study are available from the corresponding author upon reasonable request.
