# Supplementary material for: Brain gyrification in bipolar disorder: a systematic review of neuroimaging studies
Source: Brain Imaging Behav. 2022 Aug 31;16(6):2768–84. doi: 10.1007/s11682-022-00713-x (PMC9712346; doi:10.1007/s11682-022-00713-x)
Supplement: Supplementary file 1 — Supplementary file1 (DOCX 1206 KB) [file 11682_2022_713_MOESM1_ESM.docx]

Supplementary Material

**Abnormal brain gyrification in bipolar disorder: a systematic review of neuroimaging studies**

Alessandro Miola^1,2^, Giulia Cattarinussi^1,2^, Maria Lavinia Lorè^1^, Nicolò Ghiotto^1^, Enrico Collantoni^1,2,^,Fabio Sambataro^1,2^

1. Department of Neuroscience (DNS), University of Padova, Padua, Italy;
2. Padua Neuroscience Center, University of Padova, Padua, Italy

Corresponding author:

Fabio Sambataro

Department of Neuroscience (DNS), University of Padova, Italy

via Belzoni 160, I-35121 Padova

Tel: +390498211980

e-mail: fabio.sambataro@unipd.it

Contents:

Supplementary Methods

Supplementary Tables

Supplementary Figure

**S.1. Supplementary Methods**

**S.1.1. Quality assessment**

All the studies were evaluated by two independent researchers (GC, AM) for quality with the Imaging Methodology Quality Assessment Checklist (adapted from Strakowski et al, 2000) on the following parameters: subjects, imaging acquisition and analysis, and results and conclusions (Table S.1) . Overall, only one paper showed a low quality (Cao et al., 2017). All the other studies presented intermediate or high quality (see Figure S.1 for details).

**Table S.1. Imaging Methodology Quality Assessment Checklist.**

|  | **Category 1: Subjects** | **Score** (0/0.5/1) |
| --- | --- | --- |
| 1 | Patients were evaluated prospectively, specific diagnostic criteria were applied, medical illnesses were excluded and demographic data was reported |  |
| 2 | Healthy comparison subjects were evaluated prospectively, psychiatric and medical illnesses were excluded and demographic data was reported |  |
| 3 | Important variables (e.g. age, gender, intelligence quotient, i.e. IQ, handedness, socio-economic status, height or total brain measures) were checked, either by stratification or statistically |  |
| 4 | Sample size per group > 10 |  |
|  | **Category 2: Methods for image acquisition and analysis** |  |
| 5 | All neuroanatomic measurements were taken without considering group assignment or subject identity |  |
| 6 | Magnet strength > 1T |  |
| 7 | The imaging technique used was clearly described so that it could be reproduced |  |
| 8 | Measurements were clearly described so that they could be reproduced |  |
|  | **Category 3: Results and conclusions** |  |
| 9 | Statistical parameters for significant, and important non-significant, differences were provided |  |
| 10 | Conclusions were consistent with the results obtained and the limitations were discussed |  |
|  | **TOTAL** | /10 |

The score for each item ranged from 0 to 1, where 0.5 was assigned when criteria were partially met. This qualitative rating was aimed at describing the methodological limitation of published studies to help the reader in weighting the importance of the findings of a study.


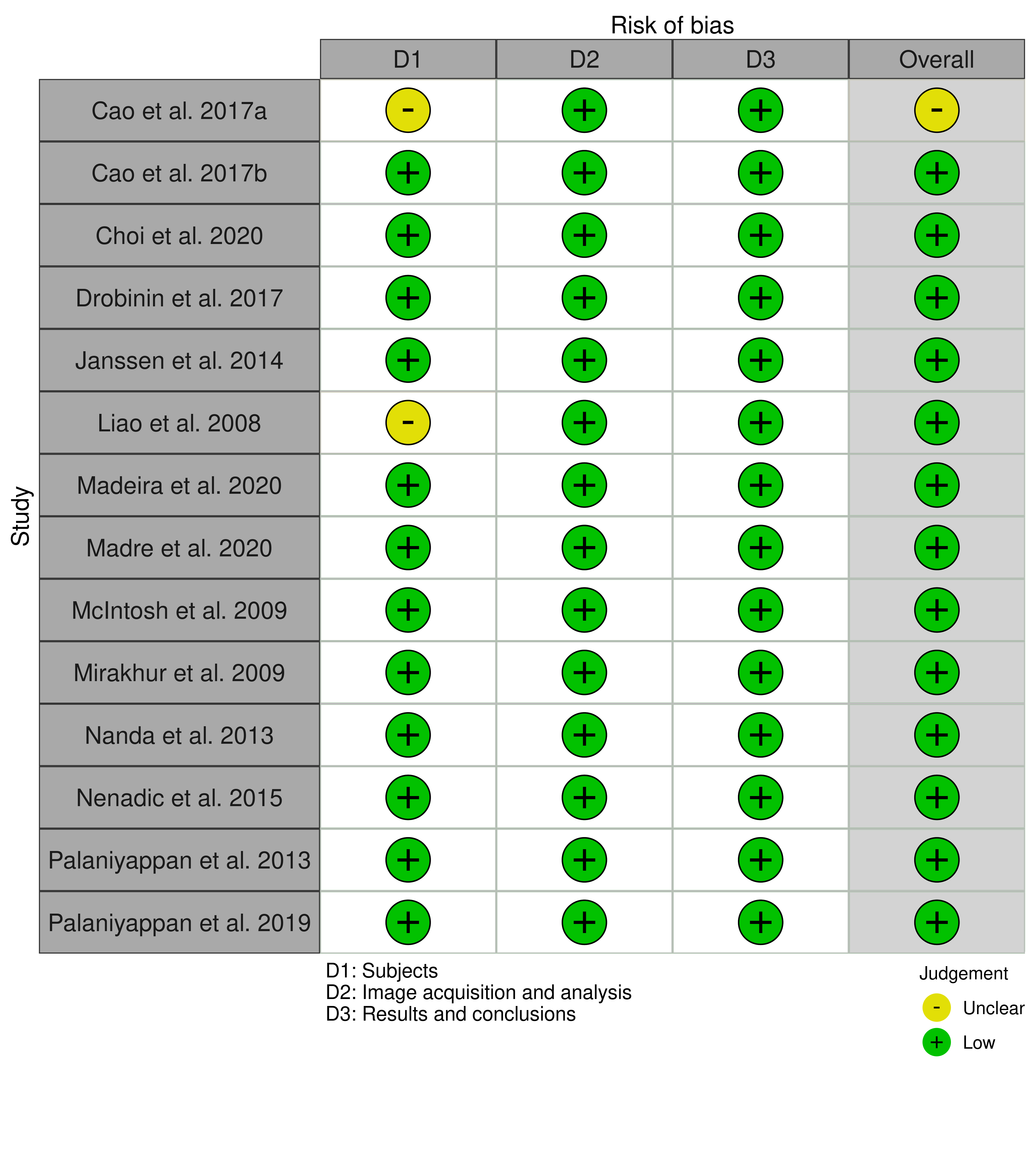


**Figure S.1. Quality assessment of gyrification studies on patients with bipolar disorders**

**Table S.2.** **List of studies excluded after full-text assessment, with reason for exclusion.**

| Thopson et al., 2016^1^ | Excluded for pooled data |
| --- | --- |
| Chakrabarty et al., 2021^2^ | No GI data |
| Jørgensen et al., 2016^3^ | No GI data |
| Patti et al. 2020^4^ | No BD population |
| Rodrigue et al. 2018^5^ | No GI data |
| Sasabayashi et al. 2021^6^ | Review |
| Sheffield et al. 2021^7^ | Excluded for pooled data |

**References:**

1. Thomson PA, Duff B, Blackwood DHR, et al. Balanced translocation linked to psychiatric disorder, glutamate, and cortical structure/function. *NPJ Schizophr*. 2016;2:16024. doi:10.1038/npjschz.2016.24

2. Chakrabarty T, Torres IJ, Su WW, Sawatzky R, Keramatian K, Yatham LN. Cognitive subgroups in first episode bipolar I disorder: Relation to clinical and brain volumetric variables. *Acta Psychiatr Scand*. 2021;143(2):151-161. doi:10.1111/acps.13245

3. Jørgensen KN, Nerland S, Norbom LB, et al. Increased MRI-based cortical grey/white-matter contrast in sensory and motor regions in schizophrenia and bipolar disorder. *Psychol Med*. 2016;46(9):1971-1985. doi:10.1017/S0033291716000593

4. Patti MA, Wochele S, Hu Y, Regier PS, Childress AR, Troiani V. Orbitofrontal sulcogyral morphology in patients with cocaine use disorder. *Psychiatry Res Neuroimaging*. 2020;305:111174. doi:10.1016/j.pscychresns.2020.111174

5. Rodrigue AL, McDowell JE, Tandon N, et al. Multivariate Relationships Between Cognition and Brain Anatomy Across the Psychosis Spectrum. *Biol Psychiatry Cogn Neurosci Neuroimaging*. 2018;3(12):992-1002. doi:10.1016/j.bpsc.2018.03.012

6. Sasabayashi D, Takahashi T, Takayanagi Y, Suzuki M. Anomalous brain gyrification patterns in major psychiatric disorders: a systematic review and transdiagnostic integration. *Transl Psychiatry*. 2021;11(1):176. doi:10.1038/s41398-021-01297-8

7. Sheffield JM, Huang AS, Rogers BP, Blackford JU, Heckers S, Woodward ND. Insula sub-regions across the psychosis spectrum: morphology and clinical correlates. *Transl Psychiatry*. 2021;11(1):346. doi:10.1038/s41398-021-01461-0
